# The ‘ethic of knowledge’ and responsible science: Responses to genetically motivated racism

**DOI:** 10.1177/03063127211063887

**Published:** 2021-12-28

**Authors:** Natan Elgabsi

**Affiliations:** Åbo Akademi University, Åbo, Finland

**Keywords:** race and racism, ethics, bioethics, population genetics, responsible research and innovation

## Abstract

This study takes off from the ethical problem that racism grounded in population genetics raises. It is an analysis of four standard scientific responses to the problem of genetically motivated racism, seen in connection with the Human Genome Diversity Project (HGDP): (1) Discriminatory uses of scientific facts and arguments are in principle ‘misuses’ of scientific data that the researcher cannot be further responsible for. (2) In a strict scientific sense, genomic facts ‘disclaim racism’, which means that an epistemically correct grasp of genomics should be ethically justified. (3) Ethical difficulties are issues to be ‘resolved’ by an ethics institution or committee, which will guarantee the ethical quality of the research scrutinized. (4) Although population genetics occasionally may lead to racism, its overall ‘value’ for humankind justifies its cause as a desirable pursuit. I argue that these typical responses to genetically motivated racism supervene on a principle called the ‘ethic of knowledge’, which implies that an epistemically correct account has intrinsic ethical value. This principle, and its logically related ideas concerning the ethic of science, effectively avoids a deeper ethical question of responsibility in science from being raised.

## The HGDP and the problem of racism

The Human Genome Diversity Project (HGDP), which was initiated in 1991 in connection with the much bigger Human Genome Project (HGP), and led by geneticist Luca Cavalli-Sforza, is the predecessor of contemporary scientific initiatives to mapping the genetic kinship and history of human populations (Cavalli-Sforza, 2005; Cavalli-Sforza et al., 1991). When it started, the HGDP was a global study of human genetic diversity that sought to explore human genetic ancestry and migration worldwide (Cavalli-Sforza et al., 1994; Human Genome Organization [HUGO], 1994). In the 2000s, however, due to severe charges of its being engaged in bio-piracy, bio-colonialism and racist typologies, the main value and focus of the project was claimed to be medical (Cavalli-Sforza, 2005). Anthropologist Linda Stone and geneticist Peter Lurquin argue that although the HGDP was groundbreaking, ‘the issue of race has placed Cavalli’s work at the center of one of the most troublesome social issues of our time. What started as an attempt to improve our understanding of human diversification and migration is now ensnared in charges and denials of racism’ (Stone and Lurquin, 2005: 170).

The charges of racism against the HGDP were made mainly in the 1990s and early 2000s, but the traces of this problem continue to haunt the evidence and the logic internal to this project. In more recent contexts, beyond strictly genetic science, interpretations and uses of the evidence of the HGDP have appeared in works of academic history and also outside of academia. In February 2015, in response to discussions about the diversity of the people of Finland, a far-right group called YleWatch used evidence that was presented in Cavalli-Sforza’s work in their racist argumentation:

The Finns’ genetically closest links are found among others in Belgium and in Northern-Germany, as the late [history-] professor Aira Kemiläinen, in her research *Suomalaiset outo Pohjolan kansa* (SKS, 1993), has written about. By contrast [to the claim to a diverse Finnish identity], we do not have even the slightest trace of dirty n****r blood in us. (YleWatch, 2015, author’s translation)

Even if it is difficult to estimate how widespread thoughts like those of YleWatch are, the utterance shows a deeply troubling connection between population genetics and racism. The central *evidence* used in this far-right contention is that the ‘Finns’ genetically closest links are found among others in Belgium and in Northern-Germany’. This evidence was originally produced by the HGDP and presented by Cavalli-Sforza as gene-frequencies between Finns, Belgians and Germans, with an ethnological intention: ‘The main value of the HGD Project lies in its enormous potential for illuminating our understanding of human history and identity’ (Cavalli-Sforza et al., 1994: 272; quote from HUGO, 1994: 1)).

The evidence was reused in the 1990s and early 2000s by Aira Kemiläinen, the most prominent and internationally acclaimed scholar on the history of racial anthropology in Finland (see Hietala, 2019), as a counterattack to Cavalli-Sforza’s claims about Finns being genetic ‘outliers’ in relation to ‘typically European’ populations (Cavalli-Sforza et al., 1994: 268–273, 1994: 268–273; Kemiläinen, 1998: 251–252, 266–271; see Anttonen, 2005; Dutton, 2008). Kemiläinen claimed that Cavalli-Sforza’s interpretation reiterates an age-old history of racism against the Finnish people by his placing the genetic origin of the Finns in Asia, together with the Sami people (the indigenous peoples of the Nordic countries). Contrary to Cavalli-Sforza, who claimed that Finns are not ‘typically European’ in a genetic sense, Kemiläinen attempted to substantiate, instead, the ‘blondness’ and typical Europeanness of the Finns by reinterpreting the HGDP data (1998; also Anttonen, 2005; Dutton, 2008; Kemiläinen, 1993; Kemiläinen et al., 1985). In her reinterpretation of Cavalli-Sforza’s scheme of gene-frequencies between human populations, she stressed that Finns are in fact genetically closest to Belgians and Germans, and not at all close to the Sami. It is this reinterpretation, as presented in Kemiläinen’s historiography, to which white supremacists refer in their attempt to substantiate the genetic ‘whiteness’ of Finns (YleWatch, 2015).

The newly crafted evidence appears in dubious argumentation about Finnish ‘blondness’ or ‘whiteness’, both under the description of being acclaimed historical research, and in racist descriptions outside of academia. It also shows that there is a problematic link between these agents that is worth considering as a serious ethical concern about scientific responsibility. Based on the same evidence, which was originally intended to have an ethnological explanatory function, actors arrive at three different conclusions:

(1) Cavalli-Sforza argues that because Finns are genetically close to the Sami people, Finns are genetically non-typical in relation to other European populations. (Cavalli-Sforza et al., 1994)
(2) Kemiläinen argues that because Finns are close to Belgians and Germans, Finns have no significant Asian genetic origin or physical character. ‘The Finns are among the blondest populations of the world’ (Kemiläinen, 1998: 273).
(3) The white supremacist YleWatch argues that because Finns are close to Belgians and Germans, ‘[We] [Finns] do not have even the slightest trace of dirty n****r blood in us’ (YleWatch, 2015).

The link between these agents puts the scientific community in the position of having to respond to Kemiläinen’s and the white supremacists’ interpretations and uses of HGDP evidence. But it also requires a response to the HGDP’s ethnological intention to compare genetic origins and identities of contemporary human groups (Foster and Sharp, 2002; Greely, 2015; National Research Council [NRC], 1997; Ramachandran et al., 2010), which starkly authorizes a typological pattern of difference and similarity between people, and which, in turn, motivates abusive and ethically abhorrent reference to genetic science (Caspari, 2010; Marks, 2002; Race Ethnicity and Genetics Working Group [REGWG], 2005; Reardon, 2004, 2011). How, then, should experts deal with racism grounded in population genetics, and what should that responsibility look like?^1^

## Ethical reflection as method

The issue of racist and abusive references to the population genetic evidence of the HGDP will be in the background of my investigation; they are not its object. The object of this study is the *scientific responses* to the ethical distress that genetically motivated racism raises for the scientific community. My questions are about how researchers and scientific communities who present and defend population genetic research that occasionally invites racism take responsibility for their research. How is responsibility recognized and dealt with by the researchers involved?

To do this, I analyse the logical structure and ethical implications of what I see as four of the main scientific responses to the problem of genetically motivated racism, responses that appear in the published discourses of initiators and ethical experts who have continuously defended the ethical value of the HGDP since the 1990s: (1) Discriminatory uses of scientific facts and arguments are in principle ‘misuses’ of scientific data for which the researcher cannot be further responsible (Cavalli-Sforza, 1997, 2005; see Pilcher, 2006). (2) In a strict scientific sense, genomic fact ‘disclaims racism’, which means that an epistemically correct grasp of genomics should be ethically justified (Barbujani and Colonna, 2010; Greely, 1999, 2015; Ho, 2010). (3) Ethical difficulties are issues to be ‘resolved’ by an ethics institution or committee, which will guarantee the ethical quality of the research scrutinized (Cavalli-Sforza, 2005; Greely, 1999; see Henderson et al., 2012). (4) Although population genetics occasionally may lead to racism, its overall ‘value’ for humankind justifies its cause as a desirable pursuit (Resnik, 1999a, 1999b, 2014).

Drawing partly on scholars who promote ‘responsible research and innovation’ (RRI), I consider responsibility as a deep ethical concern that is not limited to scientific, social or juridical responsibility (Arnaldi and Gorgoni, 2016; Bardone and Lind, 2016; Felt, 2018; Hilgartner et al., 2017). With Felt (2018: 113), I take RRI to be calling for the ‘fostering [of] researchers’ willingness and capacity to explore value-sensitive responses to the complex questions that arise at the interfaces of science and society’. Thus, beyond its being bound to merely institutional governance, RRI involves continuous conceptual reflection on the values that permeate our scientific enterprises.

The typical responses to genetically motivated racism involve mechanisms that effectively avoid raising a deeper ethical question of responsibility in science: Should a scientific enterprise, an epistemic pursuit such as the HGDP or a more specific scientific argument, generally be regarded as ethically desirable, and, if so, in what senses? I will argue that the responses to racism under investigation supervene on a principle that the biochemist and philosopher of biology Jacques Monod has called an ‘ethic of knowledge’. This means that discussions concerning the ethic of scientific research presuppose that scientific research, in its very idea, is grounded in an ethical choice, the ‘transcendental value’ of searching for objective ‘true knowledge’, which in principle ascribes ethical value to any epistemically correct account (Monod, 1971: 176; see also Beckwith, 2002: 207). I argue that the ‘ethic of knowledge’ principle restricts responsibility in science. This means, furthermore, that the typical responses to racism avoid responsibly dealing with the problem of genetically motivated racism, in terms of analyzing how population genetics contributes to significantly strengthening racist beliefs (Alper and Beckwith, 2002; Caspari, 2010; Dukepoo, 1999; Marks, 2002), and how the research disposition must be radically redirected in order to deal responsibly with that problem.

## Racism is ‘misuse’ of population genetic fact

Since the HGDP was initiated in 1991, the researchers of the project have been concerned with the risk of motivating racism with scientific findings. Henry Greely, ethical expert on the HGDP, argues that racism was one principal concern addressed during the important Alghero meeting in Italy in 1993. There, decisions were made that the HGDP should be continuously overseen by the international Human Genome Organization (HUGO), in order for the researchers of the project to concur with ethical praxis and responsible dissemination of research data (Greely, 1999; HUGO, 1994; see also HUGO, 1996).

The report from the Alghero meeting contains a response to population genetic involvement in racist argumentation that has been reiterated since then. It is the idea that any dubious interpretation or use of population genetic data is in principle a ‘misuse’ of genetic evidence for which the researcher must be vigilant. In the important Alghero report (HUGO, 1994), one reads:

Human history – and the human present – is full of racism, xenophobia, hypernationalism, and other tragedies stemming from beliefs about human populations. In the past, some of those tragedies have been perpetrated by, or aided by, the misuse of scientific information. All those involved in the HGD Project must accept a responsibility to strive, in every way possible, to avoid misuse of the project data. (HUGO, 1994: 33)

Even if it is understandable that researchers want to prevent ‘misuse of scientific information’, I would ask another sort of question: What ethical function and meaning does ‘misuse’ as a judgment usually have in scientific discourse, and particularly in the context of the HGDP? Expressing an intention to prevent ‘misuse of scientific information’ is not restricted to researchers of the HGDP; it is a widespread attitude toward ethical issues, particularly in the life sciences (Douglas, 2009; Hilgartner, 2017; McEwen et al., 2013; Pilcher, 2006; see Alper and Beckwith, 2002; Cavalli-Sforza, 2005; Greely, 1998, 2015). Thus, in order to understand the ethical function of ‘misuse’ as a judgment on racism, one must first relate the response of ‘misuse’ to distinct ideas about values in science.

The standard idea of values in science (and the value-free ideal) is that epistemic and ethical values in science are different in character. ‘[E]pistemic values delineate the goals attributed to science as a knowledge seeking enterprise’ (Carrier, 2013: 2550; also Kuhn, 1970; McMullin, 1983; Weber, 1949), whereas ethical values, for their part, are parts of judgments about the desirability of the consequences of the *application* of scientific evidence (Laudan, 1984; McMullin, 1983). In other words, the claim to value-free science is, on this account, itself upheld on a discrimination between epistemically relevant and non-relevant values (Douglas, 2009; McMullin, 1983; Weber, 1949).

This distinction opens two possibilities as to what ‘misuse’ as a scientific judgment may refer. It can refer to either failures concerning epistemic values, producing *epistemically incorrect* understandings, references, or interpretations of scientific evidence, or failures concerning ethical values, *ethically abhorrent* ways of referring to or applying scientific evidence. But these two ways of understanding ‘misuse’ actually tend to be intertwined in concrete scientific discourse, and especially in the HGDP case.

Cavalli-Sforza claims: ‘Many complex psychological factors contribute to racism, but undoubtedly a large part of the problem is generated by misconceptions, misunderstandings, and ignorance of biological facts’ (1997: 391–392). This means that ‘misuse’ as a judgment on racism, and similar statements such as racism being a ‘misconception’ or ‘misunderstanding’ of population genetics, is a judgment that racists are epistemically incorrect. The Alghero report states:

As world history includes a number of examples of racists either misusing genetic data or using the rhetoric of genetics without any real data, every possible effort must be made to minimise any misinterpretation of the analysis and plans for the HGD Project. Public attention should also be drawn to the fact that past studies on genetic diversity in human populations have actually shown that typological classification of humans into a small number of ‘races’ is scientifically invalid. (HUGO, 1994: 35)

Such statements, however, show an unavoidable ambiguity. On the one hand, racist uses of population genetics are claimed to be ‘misunderstandings’, ‘misconceptions’ or ‘misinterpretations’ of its evidence. On the other hand, they are claimed to be ideological uses of the discourse of population genetics. Is ‘misuse’, then, a mere reproach of an epistemically faulty vision or a refutation of an ethically abhorrent attitude of mind? To claim that racists ‘misuse’ genetic data is different from claiming that they simply ‘misconceive’ or ‘misunderstand’ the data. Thus, even though the ‘misuse’ response to racism is treated as a case of epistemic failure, it is implicitly a stance on an ethically abhorrent vision.

Despite this, the HGDP accounts above suggest that racism should be treated and addressed primarily as a case of epistemic incorrectness (Cavalli-Sforza, 1997; HUGO, 1994; also Cavalli-Sforza, 2005; Greely, 2015). This has further consequences. If racists’ main shortcoming is their epistemically incorrect vision of genetics, and if any epistemically correct grasp of population genetics implies that racist inferences are invalid, it means that the ethical failure of racists is understood as a consequence of their epistemically faulty vision (M’charek, 2005). As per this logic, a proper *epistemic* grasp of population genetics is thought to have *ethical value* in terms of factually undermining racism, which means that a responsible vision is directly tied to a claim to epistemic correctness (see Cavalli-Sforza, 2005; Greely, 2015). ‘Misuse’ as a judgment on racism requires the following principle:

**(P) Epistemic correctness determines the ethical value of an account**

Invoking this principle, however, would presuppose that one could easily shed off deviant interpretations and uses of science by merely referring to fact. The case described in the beginning of this study would put this simple solution into question, and the firmness of epistemic correctness is also challenged by the democratic indeterminacy of interpretation and application that is internal to science (e.g. Felt, 2018; Jasanoff, 2016, 2017; Landeweerd et al., 2015).

How, then, should epistemic correctness and incorrectness be understood? The white supremacists argue: ‘The Finns’ genetically closest links are found among others in Belgium and in Northern-Germany’ (YleWatch, 2015). In a similar vein, Kemiläinen reiterates the HGDP evidence about the genetic link between Finns, Belgians and Germans in order to substantiate her claim about the genetic Europeanness and ‘blondness’ of the Finns (Kemiläinen, 1998: 254–255). From this proposition, there is nothing in the white supremacists’ or Kemiläinen’s reference to fact that would point to its being epistemically incorrect. Even the purpose of this reference to fact correlates with the main goal of the HGDP, namely with the ethnological intention to explain the genetic character, ‘the history and identity’, of existing populations (HUGO, 1994: 1).^2^ Thus, if one looks solely at the agents’ presentation or interpretation of fact, nothing distinguishes one agent from the other. What is epistemically incorrect in the racist account compared with the other two, and to what does ‘misuse’ refer?

If ‘misuse’ cannot be determined by how agents present evidence, ‘misuse’ in terms of epistemic incorrectness must refer to considerations regarding what *conclusions* one can reasonably draw from facts (Jasanoff, 2016, 2017). Indeed, what conclusions can one draw before being epistemically incorrect? Kemiläinen’s use of the HGDP evidence in order to substantiate the ‘blondness’ of the Finns is dubious and in certain respects close to what the white supremacists’ claim, but what status should Kemiläinen’s historical work have? Despite the dubious conclusions that she draws from genetic evidence, the scientific community considers her arguments to be good and Kemiläinen to be a scientific authority (Anttonen, 2005; Dutton, 2008; Hietala, 2019). But if epistemic correctness concerns reasonable uses of population genetics, Cavalli-Sforza would have to contend that Kemiläinen ‘misuses’ these facts in order not to undermine his own claim that the HGDP does not invite prejudices or the construction of stereotypes and differences between human groups (Cavalli-Sforza, 1997, 2005). He would not want to be associated with: ‘The Finns are among the blondest populations of the world’. Kemiläinen, too, would have to claim that the white supremacists ‘misuse’ the facts that she presents in order not to concur with their racist conclusion. In this case, the *claim* to epistemic correctness and incorrectness is not a firm epistemic value but a rhetorical device used in order to fend off undesirable conclusions, while it simultaneously solidifies an idea about the inherent ethical value of epistemic correctness.

This shows two important ideas implicit to the meaning of ‘misuse’, particularly in this case of genetically motivated racism. First, there is no single unified account of what a proper epistemic grasp of population genetics is; one cannot ground a principle that epistemic correctness determines the ethical value of an account, or that epistemic correctness gives ethical justification to an account, on the presumption that population genetic evidence has inherent ethical qualities that undermine racism. Second, if the logic of principle (P) is adopted, it is, in principle, impossible to argue that a racist (or some other ethically abhorrent) reference to genetics possibly can be epistemically correct. If principle (P) is adopted, it would be impossible to argue that even an epistemically correct reference to population genetics can invite discriminatory claims.

This case regarding ‘misuse’ as a judgment on racism shows how scientific experts ascribe ethical value to epistemic value. It highlights what Monod calls an ‘ethic of knowledge’:

[H]ere is the crucial point, the logical link which at their core weds knowledge and values together – this prohibition, this ‘first commandment’ which ensures the foundation of objective knowledge, is not itself objective. It cannot be objective: it is an ethical guideline, a rule for conduct. True knowledge is ignorant of values, but it cannot be grounded elsewhere than upon a value judgment, or rather upon an *axiomatic* value. It is obvious that the positing of the principle of objectivity as the condition of true knowledge *constitutes an ethical choice and not a judgment *arrived at from knowledge, since, according to the* postulate’s *own terms, there cannot have been any* ‘true’ knowledge prior to this arbitral choice.* In order to establish the *norm* for knowledge the objectivity principle defines a *value*: that value is objective knowledge itself. Thus, assenting to the principle of objectivity one announces one’s adherence to the basic statement of an ethical system, one asserts *the ethic of knowledge*. (Monod, 1971: 176; emphasis in original)

The ‘ethic of knowledge’ asserts that scientific research has intrinsic ethical value, because scientific knowledge is in its idea conditioned by ‘the principle of objectivity’ that is not ‘arrived at from knowledge’ but is an ethical choice. This ethical choice, Monod claims, is the ‘axiomatic’ value that scientific ‘objective knowledge’ in principle is grounded in, and it is also a value that is consistently reiterated when referring to the worth of scientific knowledge (see also Beckwith, 2002: 207). It entails that any claim to presenting ‘objective knowledge’ (the claim to epistemic correctness), in principle supervenes on this ‘axiomatic’ ethical value of epistemic value intrinsic to science.

Hilgartner (2017: 52) has observed a further consequence of this ‘ethic of knowledge’ idea. Because of the ‘axiomatic’ ethical value intrinsic to science, social, political and ethical difficulties within the research disposition can never be *internal* to the knowledge-seeking enterprise, but must always be *external* and connected to the application of research. Thus, if the ‘ethic of knowledge’ is taken as a principle, it means that one guards the internal ethical value of any account *claimed* to be epistemically correct and makes that account immune from ethical consideration regarding its possible internal difficulties.

I have dwelled on the ideas surrounding ‘misuse’ in scientific discourse because understanding where an ethical problem is located, what type of problem it is and how one should deal with that difficulty, affects the very notion of what a researcher is considered to be responsible for.

In the HGDP ethics protocol from the meeting in Italy in 1993, it is argued: ‘All those involved in the HGD Project must accept a responsibility to strive, in every way possible, to avoid misuse of the project data’ (HUGO, 1994: 33). Given the above analysis, we can now better interpret what this statement means. The researcher’s responsibility – their ethical task – is to inform others ‘in every way possible’ about epistemically incorrect (and ethically abhorrent) understandings or applications of population genetics. This is what responsible conduct consists of, and what the researcher reasonably can be accountable for. But this call for responsibility is not a call for taking responsibility ‘in every way possible’ for the ethnological intentions and the typological logic internal to the knowledge-seeking enterprise if they are ethically problematic as such. The research design is regarded as internally unquestionable, for otherwise there would be no purpose of referring to an epistemically correct understanding of the project and its arguments.

The ethical function of the ‘misuse’ response shows a shallow stance regarding the relationship the HGDP has with Kemiläinen’s and the explicit racists’ ethnological endeavors, as it opens no space for scrutinizing the research-disposition’s ethnological intentions and typological logic. Given that the typological appeal is internal to the HGDP, the racist problem is not properly characterized as a mere ‘misunderstanding’, ‘misconception’ or ‘misinterpretation’ of population genetic facts. Nor is the problem of racism responsibly tackled with the reference to an epistemically correct understanding of the facts, as part of the racist problem is what the HGDP intends to explain by the means of these genetic facts (Caspari, 2010; Marks, 2002; Reardon, 2004).

If responsibility consists of pointing out claims that are epistemically incorrect, on the principle that correctness has pregiven ethical value, one not only fails to scrutinize the intentions and claims internal to the epistemic pursuit, but also outsources to other agents any responsibilities that go beyond the unclear idea of epistemic correctness (see Alper and Beckwith, 2002; Dubochet, 2008). The ‘ethic of knowledge’ principle implies that the burden of responsibility beyond epistemic correctness must be put on those who apply science; responsibility is on the ‘misusers’.

## Population genetic fact disclaims racism

The response of ‘misuse’ as a judgment on racism shows an idea that restricts, rather than deepens, responsibility in science. The judgment, I have shown, hinges on an ‘ethic of knowledge’ principle about the ethical value of scientific knowledge that guards the ethical unquestionability (or immunity) of science.

In this respect, one could turn to a closely connected response to racist uses of population genetics. In a 2005 report on the relevance of the HGDP, Cavalli-Sforza writes:

Concern that HGDP data would feed ‘scientific racism’ was also expressed by naïve observers, despite the fact that half a century of research into human variation has supported the opposite point of view – that there is no scientific basis for racism. (Cavalli-Sforza, 2005: 333)

Later in the same report he concludes:

From an ethical point of view, studies of human population genetics and evolution have generated the strongest proof that there is no scientific basis for racism, with the demonstration that human genetic diversity between populations is small, and perhaps entirely the result of climatic adaptation and random drift. (Cavalli-Sforza, 2005: 340)

This common response implies that population genetic fact has ethical value as ‘proof’ against any form of racism (Barbujani and Colonna, 2010: 291; Greely, 2015: 40; Ramachandran et al., 2010: 595; but see Reardon, 2017). Greely (2015: 40) concurs: ‘Genetic theories were used to provide support for a “scientific” racism in the first part of the twentieth century. Genomics should provide evidence against such racism’. In other words, the thought is not only that epistemic correctness determines the ethical value of an account, but that population genetic evidence as a whole undermines racism. What does this refined variant of the ‘ethic of knowledge’ entail as a response to genetically motivated racism?

The argument that genetics provides ‘proof’ against racism dwells in a traditional epistemic distinction between fact and belief, where fact is considered a scientifically substantiated belief. By this logic, there is at present no scientific evidence for racist arguments. All existing evidence disproves racist beliefs. The difficulty with this argument, however, is that *if* one were to find scientific evidence that would substantiate the inequality between populations, racism would become a scientifically justified belief (M’charek, 2005; see also Latour, 1987).

This idea may strengthen, rather than weaken, racist beliefs, because racism becomes an evidential question, exactly as the racists believe (e.g. YleWatch, 2015; see also Panofsky and Donovan, 2019). However, the problem with stereotypes and racist arguments is not that people hold on to such beliefs despite the lack of scientific evidence. Racists refer to scientific evidence despite the fact that they are often unprepared to revise their attitudes on the lack of evidence (Panofsky and Donovan, 2019). Therefore, a responsible response would make it clear that racism (like stereotypical thought) is not a question of scientific evidence, but a prejudice that science cannot demystify (Alper and Beckwith, 2002; Macer et al., 1996).

The claim that population genetics in principle has anti-racist value gives Ho, M-W (2010) from the Institute of Science in Society (ISIS) reason to ask, ‘Isn’t that just the kind of research everyone should support and applaud?’ It is not because of the intrascientific aim to deepening our knowledge of genetics, but because of the presumed extrascientific anti-racist value of genetics that ‘everyone should support and applaud’ such research. This anti-racist value is the ethical value that Cavalli-Sforza (2005) and Greely (2015) refer to as arising as a direct consequence of genomic science, which, according to Ho, means that genomics should be justified as desirable because we have no reason to doubt that anti-racist ethical value. Therefore, in the last analysis, the response that population genetics provides ‘proof’ against racism ascribes ethical justification and desirability to arguments that strengthen racist beliefs.

The claims that racists ‘misuse’ population genetics, and that genetics provides ‘proof’ against racism appear to insulate the ethnological intention of population genetics from criticism (NRC, 1997; REGWG, 2005; UNESCO, 1995; see Reardon, 2017). What the responses entail, however, are restrictions of responsibility in science. First, one locates the ethical difficulty of racism not in the *research-disposition* but in the *application* of genomic science (Hilgartner, 2017; Reardon, 2011, 2017). Second, one justifies the general *desirability* of genomic science through the idea that any epistemically correct genomic account in principle has anti-racist impact (Barbujani and Colonna, 2010; Cavalli-Sforza, 2005; Greely, 2015; Ramachandran et al., 2010). These ideas avoid the question of whether the HGDP research disposition involves undesirable elements, and thus in which senses the research should be regarded as desirable. Furthermore, the ideas avoid the question of how population genetics may contribute to strengthening racist beliefs, and how the research disposition should be subject to critique and redirection in order to deal responsibly with that difficulty.

## Racism has been ‘addressed’ by an ethics institution

The ‘ethic of knowledge’ principle about the ethical justification of scientific research can be solidified when responsibility is deflected through ethics institutions and ethics expertise. To see this, one can return to Cavall-Sforza’s claims about the ethic of the HGDP. In the report on the continued value of the HGDP from 2005, he writes:

The recommendation of the NAS-NRC [US National Research Council] committee, made public at the end of 1997, was that the HGDP could proceed, with particular attention being paid to informed consent and related ethical issues. The NIH Institute of General Medical Sciences, a chief supporter throughout, has constantly followed and revised the ethical rules of the endeavour. (2005: 334)

That the HGDP has undergone ethical scrutiny by several ethics institutions is not necessarily interesting. More interesting is what such institutional supervision and approval is supposed to mean in the context of the ethical difficulties of genome diversity research.

The 1997 NIH report to which Cavalli-Sforza refers above highlights one question as fundamental to any ethical evaluation of research: ‘[T]he most important ethical question in research is always whether the research is worth doing’ (NRC, 1997: 59). This statement is important because it suggests that the function of ethics institutions is to determine the worth of research. Thus, Cavalli-Sforza’s purpose of highlighting that ethical, legal and social issues were ‘addressed’ in the project, and ‘subsequently’ reviewed by an ethics advisory committee that was approved by the US National Institutes of Health (NIH) Institute for General Medical Science (Cavalli-Sforza, 2005: 336), is, it seems, to justify the ethical worth of the scientific pursuit against the accusation that it feeds racism (2005: 340). This was a main reason why his project had to undergo supervision by the NIH ethics committee in the first place (NRC, 1997; also M’charek, 2005). Hence, Cavalli-Sforza thinks that the NIH supervision justifies the ethical value of the epistemic pursuit of seeking knowledge about human genetic variation for possible ethnological utility (see Felt, 2018; NRC, 1997). In this procedure, the methods for reaching an epistemic goal, as well as the goal itself, are evaluated. In the case of positive evaluation (as was the case with the HGDP), the goal is justified as desirable, by the means of institutional conclusions asserting that genomics ‘falsifies’ typological thought and shows that racial thinking is ‘invalid’ (HUGO, 1994: 35; NRC, 1997: 56–59; see Cavalli-Sforza, 2005; Greely, 2015). Nevertheless, the most important role of invoking NIH ethical supervision is its guarantee that the genomic pursuit can ‘proceed’ (Cavalli-Sforza, 2005: 334), because the institution has authoritatively ‘determined that the HGDP is worth pursuing’ (Resnik, 1999b: 16).

It is not the content of the NIH verdict, but the purpose and function of referring to NIH ethical supervision that becomes a response to racism. Because of the ethical unease of the charges of racism, bio-piracy and bio-colonialism surrounding the HGDP in the 1990s, which led to a grand evaluation of the enterprise by the NIH, it is reasonable to argue that researchers regard the ethics institutions that have ‘approved’, ‘subsequently reviewed’ and decided that the project can ‘proceed’ as guaranteeing the ethical worth of the epistemic pursuit (2005: 334, 336; see also NRC, 1997; Resnik, 1999b). In this light, Greely expresses the same understanding of the meaning of ethical supervision as he responds to scholars charging the pursuit with having racist dispositions:

And so, four years after its completion, the HGDP’s Model Ethical Protocol [NAmC, 1997] has been largely ignored. The HGDP is accused, without proof, of things that it is committed to avoiding, and … is urged to address issues about which it already had detailed, innovative, and progressive positions. (Greely, 1999: 299)

Furthermore, in response to the plea for further ethical reflection on the enterprise, because it was involved in discriminatory argumentation (e.g. Alper and Beckwith, 1999; Cunningham, 1998; Harry and Dukepoo, 1998; Rural Advancement Foundation International [RAFI], 1993; UNESCO, 1995), Greely stresses ‘the fact that the HGDP has been addressing those issues’ persistently throughout the years, in institutions and working-groups (1999: 297).

This reference to the function of ethical supervision constitutes a third typical response to genetically motivated racism. It consists of the idea that ethics institutions, programs or working groups, have already ‘addressed’ any ethical issues of racism that the scientific pursuit involves (Greely, 1998, 1999, 2001, 2015). Moreover, since having ‘addressed’ the issues of racism, the ethics institutions have allowed for, and even encouraged, the further proceeding of the pursuit from the viewpoint of an area of ‘ethical expertise’ (Hilgartner et al., 2017: 834; also Jasanoff, 2011; Reardon, 2011).

Bardone and Lind (2016) have called this a ‘technocratic’ logic regarding the relationship between the epistemic and the ethical domains. It involves at least three strong presuppositions about ethics:

(1) Ethics is treated as a reflexive add-on, ‘something externally added to the pre-existing pursuit’ (Bardone and Lind, 2016: 9; also Felt, 2014).
(2) Ethics is understood as an exclusive area of expertise, separated from the area of technical or epistemic expertise. Ethics ‘experts’ handle ethical difficulties connected to the epistemic ‘goal’ of the pursuit, by finding solutions and making decisions to reach that epistemic ‘goal’ (Bardone and Lind, 2016: 9–10; Felt, 2018; Hilgartner, 2017, 2018).
(3) Ethics cannot concern the technical matters regarding the epistemic ‘goal’ of a knowledge seeking enterprise. ‘[T]here is no way to steer or influence the pursuit internally, because that would mean to interfere with the accomplishment of goals, which is inherently a technical issue’ (Bardone and Lind, 2016: 9). For example, even though the NIH evaluated the HGDP enterprise and claimed that one risk with its research design was its typological structure, the expert inspection did not interfere with the HGDP goal to explain ‘human history and identity’; it was left intact (NRC, 1997). This too suggests that ethical issues are typically understood as extrascientific issues; namely as ‘implications’ or consequences related to the application of research (Hilgartner, 2017, 2018; also Alper and Beckwith, 2002; Dubochet, 2008; Jasanoff, 2016).

This ‘technocratic’ understanding of ethics is generally strong in the life sciences. Regardless of whether one looks at clinical health care or genomics, the *logical purpose* of ‘ethical expertise’ is pragmatically to remove ethical obstacles by ‘resolving’ how to eliminate them, in order to provide ‘ethically justified’ options and decisions (Adams, 2013; Tarzian et al., 2013: 4–5; see Bardone and Lind, 2016). The goal of Ethical, Legal and Social Implications (ELSI) programs, to take an example, is said to be ‘to identify and resolve the ethical, legal and social issues raised by the [genomic] project[s]’ (Cho et al., 2008: 5; also Hilgartner et al., 2017; NRC, 1997; Resnik, 1999b; Roth and Yesley, 1992). Even critics of ELSI (and ELSA in the EU) institutions or working groups often concur with this pragmatic solution paradigm, as ethical issues are envisioned as problems to be ‘solved’ by a group of experts (Henderson et al., 2012; McEwen et al., 2014; see Felt, 2018).

This understanding of ethics infers that ‘ethical expertise’ means having authority over ethical matters in the sense of stipulating what is legitimate to think and decide regarding any non-epistemic problems that arise in relation to a scientific pursuit and its application (Adams, 2013; see also Hilgartner, 2017; Hilgartner et al., 2017; Hilgartner, 2018; Reardon, 2011). However, it also suggests that ethical issues in general are understood as analogous to *technical* obstacles that must be removed because they interfere with the accomplishment of the epistemic goals set inside the scientific pursuit (Bardone and Lind, 2016).

The full circle of the ‘technocratic’ logic, then, is that one outsources ethical issues to an area of ‘ethical expertise’ that should ‘resolve’ such issues to justify the further proceeding of the scientific pursuit with reference to the fact that one has ‘addressed’ those issues.

Nevertheless, this ‘technocratic’ idea of ethical institutionalism constitutes a problematic take on genetically motivated racism, because it displaces internal concerns that should, in light of the reflexivity of RRI, belong to responsible science. If the role of ‘ethical expertise’ is to help reach the goals of the epistemic pursuit, one presupposes that the goals are ethically desirable. This locates any ethical difficulties not in the research disposition (in the aims and methods of a knowledge seeking enterprise), but in the possible application of research. Responsible research becomes a matter of managing those applications by the means of ethical recommendations set inside ethics institutions, with the epistemic pursuit left intact. Consequently, if the purpose of ethics institutions must coincide with reaching an epistemic goal, then revision of epistemic values is not considered a matter of *ethical*, but rather of *technical* scrutiny (Bardone and Lind, 2016; see NRC, 1997).

But there is a further consequence of this idea. If ethics is envisioned in a ‘technocratic’ way, as the handmaiden of the epistemic search for knowledge, it means that ‘ethical expertise’, as often understood in science, is not a matter of ethics at all, but just another epistemic matter.

Thus, from an ethical point of view, this ‘technocratic’ idea of ethics and ‘ethical expertise’ is superficial, since any proper understanding of ethics and responsibility presupposes not that ethics merely accompanies any epistemic concerns, but that epistemic matters must be judged against what is *good* in the context of a human lifeworld. This is where the deepest conflict between science and ethics transpires.

## Population genetics is for the greater good of humankind

Does this mean that the epistemic concern with finding *truth* has precedence over the ethical concern with what is *good*? A fourth typical response to genetically motivated racism must be understood from within this temptation to think that the desire for knowledge, which guides any epistemic aspiration, is analogous to, or even replaces, what is humanly good (David, 2001: 153; Radder, 2017). As David explains:

Epistemology treats justified belief somewhat like ethics treats right action: Holding a justified belief is like holding a belief in the right kind of way. Truth is treated in analogy to *the good* – truth is, as it were, the good as far as epistemology is concerned. (David, 2001: 154)

Although the desire for knowledge resembles the good as far as the theory of knowledge is concerned, it does not mean that scientific concerns with truth, and the facts and justifications presented in the name of scientific truth, of necessity are good. Henri Poincaré wrote: ‘The search for truth should be the goal of our activities; it is the sole end worthy of them’ (1907: 11). Similarly, the ‘ethic of knowledge’ that Monod fleshes out is grounded in the contention that knowledge is a ‘transcendental value’; not an epistemic value, but a transcendental good (Monod, 1971: 178).

If one looks to concrete scientific and bioethical discourse, scientific knowledge is often considered to be both an ideal and pragmatic good in the sense that it deepens humankinds’ cumulative knowledge so that the human lifeworld may benefit from the possible innovation that it brings (Radder, 2017; Rotblat, 1999; also NRC, 2003; Resnik, 2014). The social responsibility of any scientist, says bioethicist Resnik (2014: 188), is to ‘engage in activities that enhance or promote the common good’, which is achieved, in part, by ‘conducting research which benefits the public’. Pragmatically, as Joseph Rotblat’s claims, ‘scientific research is very likely to bring further benefits to all of us, and we should not do anything that may hinder such outcomes’ (1999). Should one hold this idea, it seems that ‘truth’ is not as a mere epistemic imitation of the good, but has replaced it.

This ‘transcendental value’ of scientific knowledge permeates the fourth typical response to genetically motivated racism. In a 1999 response to the kind of racism (including bio-piracy and bio-colonialism) that the HGDP has been accused of, Resnik evaluates some ethical implications of the project and writes:

Scientists who study different races do need to be aware of the social and political context of the research – science is not value free – but they do not need to stop their research because it might have harmful consequences. Although scientists should sometimes refrain from research because it could have harmful consequences, scientists and the public need to balance potential benefits against potential harms in assessing scientific research. The HGDP’s potential benefits for all human beings, and particularly indigenous populations, outweigh its risks. (Resnik, 1999b: 17)

Resnik argues that ‘[o]ne of the main justifications of the HGDP is that it will help provide researchers with a more complete and comprehensive understanding of human genetics’ (Resnik, 1999b: 15), which means that the search for knowledge of human genetics is in itself the very value that *justifies* the epistemic pursuit. In fact, no further justification is provided, only inferences that ‘the HGDP will also help scientists learn about human evolution, genetic relationships between different human populations, human migration patterns, relationships between human languages, and human health and disease’ (1999b: 16; also 1999a). Thus, the search for knowledge about human genetics is as such considered to be an intrinsic good, with further ‘potential’, he says in the quote, for application in other scientific contexts and in society at large.

But it is only against the presupposition that scientific knowledge has this strong transcendental value that one can make sense of Resnik’s conclusion that ‘[t]he HGDP’s potential benefits for all human beings, and particularly indigenous populations, outweigh its risks’. Indigenous peoples themselves, those who were humiliated, exploited and objectivized in the project, do not concur with Resnik’s description (Cunningham, 1998; Harry and Dukepoo, 1998; Marks, 2005). Thus, Resnik refers to a value that overshadows experiences and self-understandings. If Resnik’s bioethical reflection would not presuppose that scientific search for knowledge is a good in terms of having ‘potential benefit for all human beings’, the population genetic pursuit would have to be evaluated on completely other grounds. It would have to be evaluated as involved in racist argumentation, and it could not be saved by referring to an ideal about the transcendental value of scientific search for truth (see Garrison et al., 2019). There is no meaningful claim to population genetics having ‘potential benefits for all human beings’ without simultaneously presupposing the transcendental value of scientific knowledge as the sole good end (see Poincaré, 1907).

A further implication of Resnik’s thought is his insertion of the transcendental value of scientific knowledge into a utilitarian risk calculation about the ‘possibility’ for good and bad consequences. Resnik argues that ‘scientists and the public need to balance potential benefits against potential harms’, which presupposes that one actually *could* weigh, say, racist inferences, exploitation and humiliation of indigenous peoples and potential medical inventions, in a utilitarian calculation of benefit and risk (see Jasanoff, 2016). This calculation would forthwith determine whether the epistemic pursuit is ethically *justified*, in the sense of ‘worth pursuing’ (1999b: 16; also NRC, 1997: 56–59). Tautologically enough, Resnik’s conclusion that the HGDP’s ‘potential benefits’ for humankind ‘outweigh its risks’ already presupposes that the value of scientific knowledge, i.e. claims to deepening our understanding of human genetics and its pragmatic ‘potential’ innovative spin-offs, is an extremely powerful transcendental value that ‘outweighs’ other factors. In Resnik’s risk-calculation, this value and its ‘potential benefits’ ‘outweighs’ all racism, exploitation and humiliation (as well as bio-colonialism) that the HGDP evidently has been involved with and still upholds (Bradby, 2006; Dukepoo, 1999; Harry, 2008; Marks, 2002, 2005).

The ethical difficulty with this idea is that even if one ideally treasures the value of our epistemic search for truth the way Resnik does, one needs a strong normative commitment in order to think that ethics can be responsibly treated as calculation of benefit and risk, or that the transcendental good of knowledge ‘outweighs’ any other ethical concerns (see MacLean, 2005). This normative position could be criticized in two ways for overlooking the seriousness of genetically motivated racism.

First, risk-calculation restricts the idea of scientific responsibility, because the HGDP involvement with racism is treated as a case of ‘dual use research’, or as a ‘dual use dilemma’. It is treated as a large-scale biotechnology that should intentionally benefit society but occasionally may be abused (NRC, 2004: 14–15; Shamoo and Resnik, 2015: 294). On such large-scale levels, scientists should be aware of, as Resnik puts it, ‘the potential for racist implication of their work and racial biases in their study design’ (1999b: 17). This ‘awareness’ regarding positive and negative impact of science is what scientific responsibility, on this account, is thought to consists of. Being ‘aware’ of the ethnological intentions and typological logic internal to the enterprise is applauded, but, as Resnik stresses, this awareness does not mean that scientists need to take action regarding those internal difficulties (Bardone and Lind, 2016; Felt, 2018). Thus, one could question how responsible this response is.

Second, risk-calculation restricts the idea of bioethical responsibility, because risk-calculation is thought to be a responsible way of scrutinizing ethical concerns in science. Resnik apparently thinks that a responsible bioethical discourse consists of merely describing benefit and risk, where racism is a minor negative impact in this calculation, and the transcendental value of scientific knowledge is a major positive one. In this discourse, the values are comparable, and the one ‘outweighs’ the other. Even if prediction of risk, on the political, juridical or institutional level, is imperative when evaluating and managing science and innovation that may have large-scale social impact (Felt, 2018; Jasanoff, 2016), it is important to emphasize the risk of supporting a historically persistent attitude of mind that refines ethnological and typological ways of reasoning. This problem is not responsibly tackled with the claim that the transcendental value of scientific knowledge ‘outweighs’ that distress.

Proponents of RRI contend that ethical consideration and responsibility in science must not leave aside the large-scale political, juridical or institutional aspects of science and innovation that ELSI and ELSA institutions traditionally deal with; it needs only to involve more in-depth reflection on particular practical matters (Felt, 2018; Hilgartner et al., 2017; Rip, 2014). It is important, however, that these practical matters are not evaluated against epistemic, institutional or juridical criteria as to what ethics and responsibility in science consists of, but against the concept of care for what is good for other beings (Bardone and Lind, 2016). In this analysis, I have shown some of the consequences of anchoring ethics and responsibility in principles and concerns astray from what is humanly good. In order to see what is good, and raise the deep question of responsibility in science, one needs to break the strong ‘ethic of knowledge’ principle regarding the a priori ethical value of scientific knowledge, its closely connected ‘technocratic’ relative that ethics merely accompanies any epistemic aspirations, as well as ultimately with the presumption that knowledge is a ‘transcendental value’ that has replaced what is humanly *good*.

## Conclusion

My analysis shows that the typical responses to problems of racism derived from the HGDP presuppose an ‘ethic of knowledge’ principle that in different ways restricts questions of ethics and responsibility in science to extrascientific concerns. The main distinction in these responses consists of a categorical watershed between the epistemic realm and the ethical one. Paradoxically enough, however, the epistemic domain is consistently treated as an ethically justified good, immune from further ethical scrutiny.

Treating the epistemic domain as intrinsically ethically justified is itself ethically problematic as it avoids the unease regarding how the ethnological intentions and typological logic internal to the HGDP enterprise invites, and even strengthens, the racist beliefs. By contrast, the principle gives ethical justification to pursuits that involve those typological difficulties. The normative conclusion of this study is that the ‘ethic of knowledge’ idea about the ethical value of the epistemic domain needs to be more thoroughly reconsidered in order to deal responsibly with the ethical meaning of science as a concrete part of society.

## Notes

1. This difficult link between population genetic arguments and racism is observable also in other empirical contexts, for example in cases of far-right claims to ‘indigenous Britishness’ (Brubaker, 2015; Fortier, 2012). In this study, I analyse the structure of the responses to a problem that may also be empirically apparent in contexts other than the ones I exemplify.

2. One possible misunderstanding of population genetics on which both Kemiläinen and the white supremacists seem to lean is the idea that the population estimates used within the HGDP correlate with ethnic identity concepts in ordinary life. Population geneticists have frequently pointed out that while ancestry is a natural fact, racial and ethnic markers are social imaginations that in principle cannot be rooted in the facts about ancestry that population genetics show (Bliss, 2012; Foster and Sharp, 2002; Smart et al., 2012). This radical separation between the natural fact of ancestry and the construction of racial and ethnic identities, however, is a contested claim, and belongs to one of the perennial problems of population genetics that occasionally goes beyond the scope of this paper (Caspari, 2010; Duster, 2005; Panofsky and Donovan, 2019).
